# Selective and Tunable Routes for Glucose to Fructose Conversion Using MgCl_2_ Catalysis and Comparison to Other Metal Ions

**DOI:** 10.1002/open.202500495

**Published:** 2026-02-01

**Authors:** Ramesh Maragani, Sebastian Meier

**Affiliations:** ^1^ Department of Chemistry Technical University of Denmark Kgs Lyngby Denmark

**Keywords:** fructose, glucose, hydride shift, NMR, speciation

## Abstract

The conversion of glucose to fructose is an important step for the formation of biofuels, fine chemicals and in the food industries. Mg^2+^ is the most abundant divalent cation in living cells and sea water and could be an environmentally friendly biomimetic catalysts for glucose‐to‐fructose isomerization in water, while holding relevance to prebiotic chemistry. Here, we demonstrate that the catalytic performance of MgCl_2_ can be tuned using strategies that limit the presence of basic oxide. Upon calcination and reaction under N_2_, glucose isomerization in water at 120°C approached the thermodynamic equilibrium (≈42% fructose) within 30 minutes. Isotope tracking showed that the isomerization proceeds via competing pathways. Compared to Al^3+^ and Cr^3+^, the stereoselectivity is considerably lower for Mg^2+^ than for Al^3+^ and Cr^3+^. Effects of formic acid on the initial rate of glucose‐to‐fructose isomerization showed a slowing of the reaction catalyzed both by Mg^2+^, Al^3+^, and Cr^3+^. Inhibition decreased in this order, which resembles decreasing p*K*a values of the metal ions in aqueous solution. Hydrolysis of aqua ions appears to generate active species for the 1,2‐hydride shift in all cases, where the formation of transient and non‐specific interactions between Mg^2+^ and carbohydrate results in a moderate stereoselectivity.

## Graphical abstract

MgCl_2_ is a cheap catalyst for glucose to fructose conversion in water with excellent selectivity if competing pathways leading to C—C bond breakage avoided by control of pH and oxygen and use of mild temperatures. Both a stereoselective 1,2‐hydride shift and isomerization via an enediol intermediate contribute to the isomerization. Similarities and differences are discussed to Lewis acidic ions that are popular in sustainable chemistry.

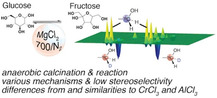

## 1 Introduction

Magnesium is abundant in nature and accounts for 2% of the Earth's crust. Accordingly, Mg^2+^ is ubiquitous in biological systems and is the most abundant divalent cation inside cells, where it predominantly coordinates to phosphate groups and to proteins, while being soluble near neutral pH and inert to participation in unwanted redox chemistry [1]. Thus, magnesium is a cofactor in more than 300 enzyme systems, including enzymes for the isomerization of glucose to fructose [2, 3, 4]. Mg^2+^ salts are therefore plausible catalysts for bio‐inspired processes and for understanding the evolution of enzymatic processes, not least considering the abundance of Mg^2+^ in seawater (≈50 mM). Due to their abundance in nature, magnesium salts could be cheap and widely available catalysts that do not pose high risks of resource depletion or environmental footprint [5, 6, 7]. At the same time, Mg^2+^ salts often operate effectively under near‐neutral pH and are well suited for redox‐neutral processes such as isomerization, due to the low propensity of Mg^2+^ to participate in electron transfers.

Magnesium ions stabilize the enzyme and enhance its activity in enzymatic mechanisms of glucose isomerization [3, 4, 8, 9]. Alkaline earth metal ions including Mg^2+^ are also known to coordinate to carbohydrates in water near the reducing end through transient and non‐specific interactions [10, 11, 12, 13], hinging on the Lewis acidity of Mg^2+^, which is comparable to the Lewis acidity of divalent first‐row transition metals and may aid in avoiding side reactions that may occur for catalysis with stronger Lewis acids [14]. Accordingly, the direct use of magnesium oxide [15, 16, 17, 18, 19, 20], magnesium halides or more advanced magnesium‐containing materials [21] in the glucose‐to‐fructose isomerization reaction is recently gaining interest [22, 23]. This isomerization is central in the food industries and in the conversion of glucose to fructose and onward to biofuels and fine chemicals [24, 25, 26, 27, 28]. Thus, the conversion of glucose to fructose affords the subsequent formation of precursors such as furanic compounds and lactic acid. Mg^2+^‐based materials have therefore been used for the conversion of glucose to furanic compounds via fructose as the likely intermediate [29, 30, 31]. Hence, a challenge is to identify conditions that favor the intramolecular isomerization via CH bond cleavage and H‐transfer, while at the same time leaving the carbon backbone intact and avoiding dehydration.

Using natural or synthetic magnesium salts as the catalysts, previously reported fructose yields reached on the order of 36% with selectivities of 80%–85% when using MgO (alone or in mixed oxides) [15, 32, 33] or MgCl_2_ as the catalyst [22]. Similarly, the glucose to fructose conversion with MgBr_2_ achieved a fructose yield of 32% and selectivity of 72% [23], while initial work preferred CaCl_2_ and typically resulted in fructose yields of less than 31% [34]. Recently, yields of 29.8% with a selectivity exceeding 90% have been reported for MgSnO_3_ material with a balanced acidity and basicity [21]. Inspired by this work, we herein evaluated the possibility of rapidly reaching equilibrium distributions of glucose, fructose, and mannose with an expected fraction of fructose near 42% using MgCl_2_, by tuning the reaction system to avoid competing reactions leading to the degradation of the carbon backbone in hexoses.

Little focus has hitherto been placed on the understanding of the competing pathways for glucose conversion in the presence of magnesium salts, although insight into the underlying key mechanistic details could support the optimization of materials and processes for efficient and efficacious glucose‐to‐fructose conversions. Specifically, the nature of the catalytically active species in aqueous solution remains elusive, as does the relevance of different pathways, specifically Lewis acid catalyzed hydride shifts and Brønsted base catalyzed Lobry de Bruyn–Alberda–van Ekenstein transformations via enediol intermediates. Recently, gas phase data were used as indication that [MgCl]^+^ species, rather than hydroxide forms, could elicit a hydride shift in the MgCl_2_‐catalyzed glucose‐to‐fructose isomerization [22]. Here, we use direct isotope tracking methods to identify elusive details in the stereoselectivity of Mg^2+^, Al^3+^ and Cr^3+^ catalyzed glucose‐to‐fructose isomerization in water. Compared to Al^3+^ and Cr^3+^, the formation of less rigid complexes between substrate and catalyst is found to elicit low stereoselectivity (≈70% to 30%) in 1,2‐hydride shifts for Mg^2+^ catalyzed via transient non‐specific interactions. Isomerization using MgCl_2_ proceeds both via enediol species and hydride shift, where the relative contributions can be tuned via the addition of Brønsted acids. Functional studies, including such titrations with acids or halides into the reaction medium, are suggested, indicating that hydrolysis of aqua ions contributes to forming the active species for the hydride shift.

## 2 Results and Discussion

### 2.1 Effect of Calcination Under Nitrogen on Selectivity

**FIGURE 1:**
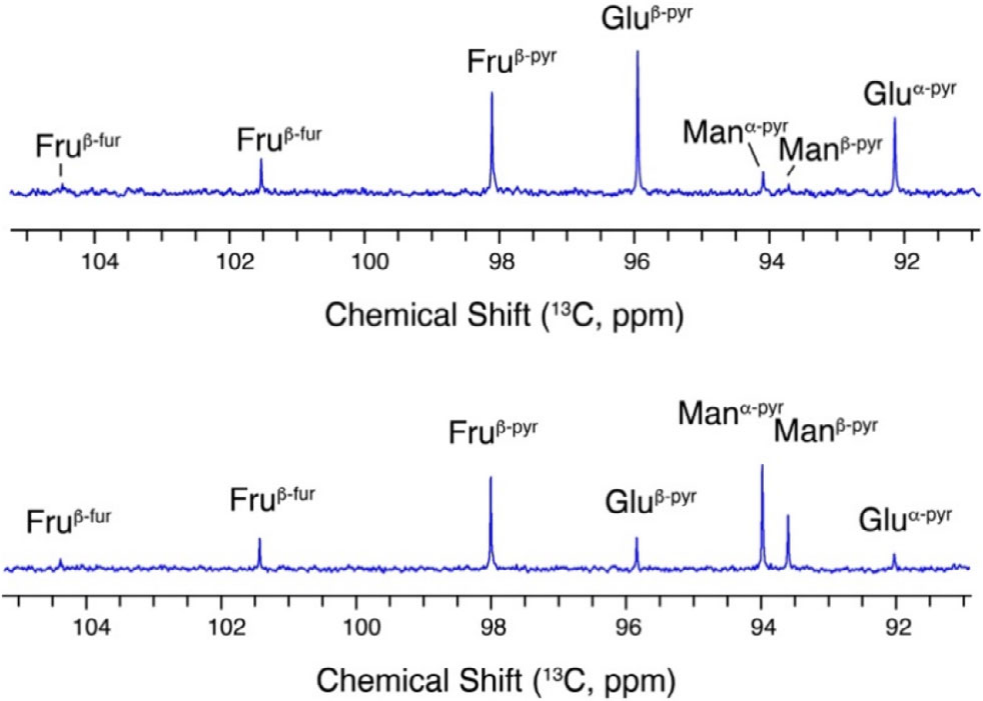
QNMR spectra (1D ^13^C) for the carbohydrate mixture formed from glucose (top) or mannose (bottom) at 120ºC after 30 min, using the anomeric hemiacetal and hemiketal sites as structural reporters.

**SCHEME 1:**
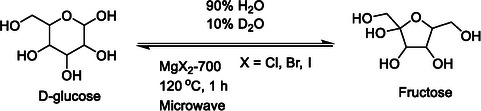
Glucose to fructose conversion using magnesium halides that were calcined at 700°C.

We initially took inspiration from recent work [22] describing the conversion of glucose and fructose at 120ºC in a microwave reactor using Mg^2+^ salts as the catalyst in water (Scheme 1). Isomerization reactions were analyzed using nuclear magnetic resonance (NMR) spectroscopy, owing to the strengths of high‐resolution NMR in distinguishing carbohydrate isomers [35, 36, 37]. Quantifications were performed using qNMR (Figure 1), where one‐dimensional ^13^C NMR spectra were acquired with an inter‐scan relaxation delay of 60 s and inverse‐gated decoupling, prior to integration of resolved carbon sites [38]. Our initial screening results indicated that MgCl_2_ that was calcined at 700°C was preferable relative to MgCl_2_ that had not been calcined or to MgCl_2_ that had been calcined at lower temperatures (150°C). MgCl_2_ was preferable compared to MgBr_2_ or MgI_2_. The latter halides had a bigger propensity than MgCl_2_ to release small acids as byproducts rather than fructose during standard reaction conditions at 120°C. This behavior is consistent with a higher stability of Mg^2+^ salts with smaller halides compared to salts with bigger halides (Figure S1). Considering this trend in stability, all further procedures focused on MgCl_2_ as the catalyst.

**FIGURE 2:**
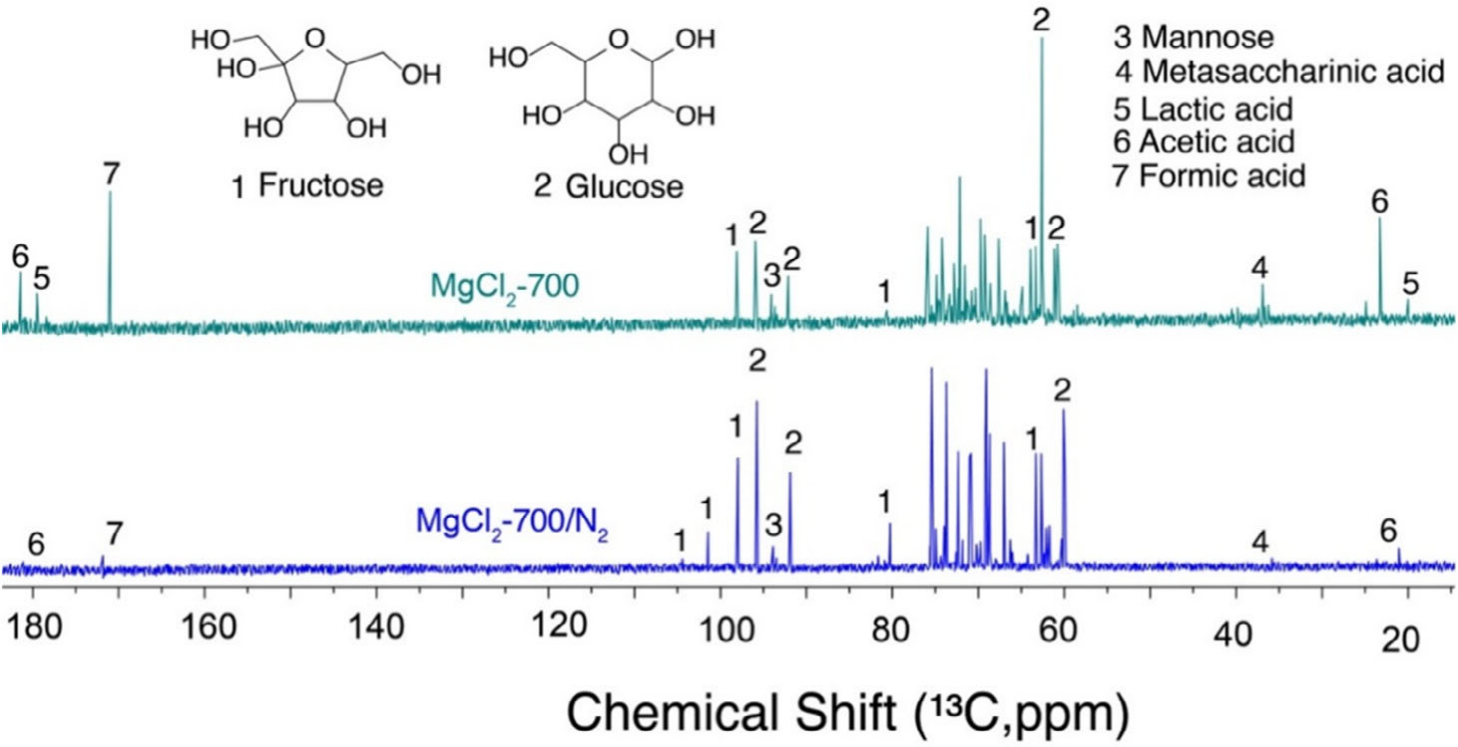
^13^C NMR spectra of glucose to fructose conversion using MgCl_2_ calcined at 700°C in air (top) and under nitrogen (bottom). Reaction conditions: 0.1 g (1 equiv., 0.55 mol/L concentration) of glucose, 0.02 g (0.38 equiv., 0.21 mol/L concentration) of MgCl2‐700/air (above) and 0.02 g (0.38 equiv., 0.21 mol/L concentration) of MgCl_2_‐700/N_2_ (bottom), 1 mL solvent (0.9 mL H_2_O, 0.1 mL D_2_O) and stirring for 1 h in microwave at 120°C.

Initial experiments in this setup showed that byproducts including formic acid, acetic acid, lactic acid, and metasaccharinic acids accounted for ≈21% carbon balance and limited the yield and selectivity in the formation of fructose even when using the preferred magnesium halide, MgCl_2_ calcined at 700°C (Figure 2). Considering that the catalytic propensity of the catalyst appeared to correlate with its stability during calcination for different magnesium halides, we hypothesized that calcination should exclude the risk of producing spurious amounts of MgO or should remove residual MgO species. Hence, we chose to pursue calcination of MgCl_2_ at 700°C under a nitrogen atmosphere. Upon calcination at 700°C under N_2_ (yielding catalyst material that is henceforth termed MgCl_2_‐700/N_2_), we found a considerably improved selectivity of glucose‐to‐fructose isomerization using this catalyst. X‐ray diffraction analysis validated that minor changes without impact on the framework occurred during calcination at 700°C under N_2_ (Figure S2). Using (MgCl_2_‐700/N_2_), selectivities in the isomerization to fructose and mannose transgressed 88% on a carbon basis, with formic acid, acetic acid, and metasaccharinic acid as the only detectable organic byproducts. We hence concluded that the absence of MgO species in the MgCl_2_‐700/N_2_ catalyst is conducive to a highly selective isomerization reaction using dissolved magnesium halide in water. This interpretation is consistent with previous findings of a complex pathway of sequential dehydration and hydrolysis toward partly hydrated MgOHCl and MgO species in air [39]. Overall, these data indicated that tuning the acid and base strength and purifying the MgCl_2_ via high temperature calcination under N_2_ is essential for controlling selectivity, favoring fructose over side products.

**SCHEME 2:**
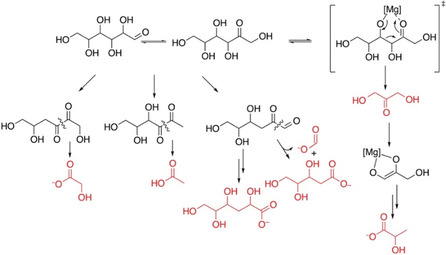
Schematic depiction of the plausible conversion pathways toward the molecular species that were detected in postreaction material of the MgCl_2_ catalyzed conversion of glucose.

The formation of formic acid, acetic acid, lactic acid, and metasaccharinic acids could be reduced approximately by one order of magnitude, when excluding oxygen from the calcination step and the catalyst material was kept under N_2_ atmosphere for further reaction and storage. An initial time point taken after 10 min of reaction showed that dihydroxyacetone had formed, prior to the formation of lactic acid (Figure S3A). This observation indicates that the MgCl_2_‐based reaction system can catalyze a pathway that resembles Embden–Meyerhof–Parnas glycolysis, as it proceeds via dihydroxyacetone from fructose through retro‐aldol cleavage, prior to dehydration to methylglyoxal and conversion to lactate in a Cannizzaro reaction. Plausible pathways to the detected byproducts are schematically depicted in Scheme 2. These byproducts resemble products formed in the presence of alkaline metal oxides such as Ba(OH)_2_ and further indicate that a procedure that minimizes the formation of metal oxides is vital for ensuring the highly selective conversion of glucose to fructose [40]. Isotope tracking data using [1‐^13^C] glucose validated the fate of the C1 position in the formation of byproducts. This position was predominantly converted to formic acid, or it was converted to the C1 position of metasaccharinic acids, the C2 position of glycolic acid and acetic acid, as well as the C1 and C3 position of lactic acid (Figure S3B).

**FIGURE 3:**
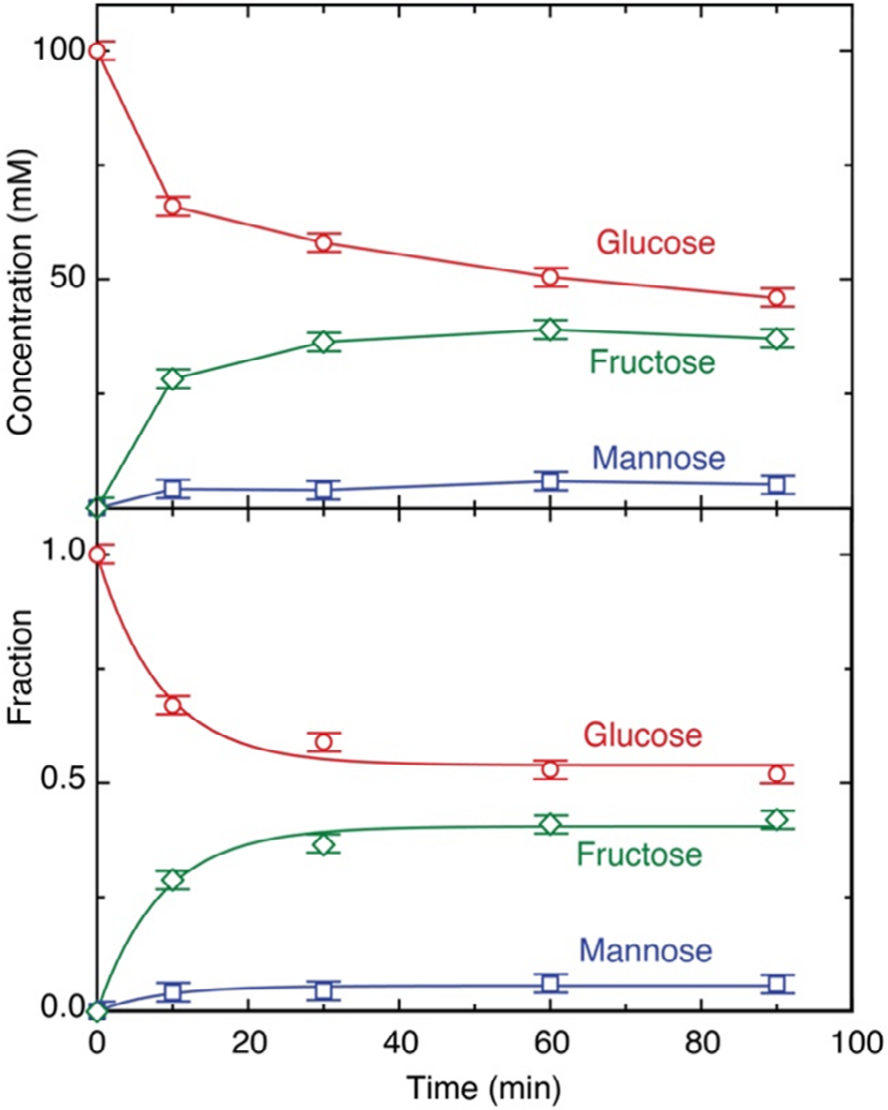
Time course of glucose, fructose and mannose concentrations as determined by quantitative ^13^C NMR showing that maximum concentrations above 40% fructose can be reached within 1 h, while byproduct formation reduces the maximum yield at higher reaction times (top). The fraction of glucose, fructose, and mannose indicates an approach of equilibrium with a first order rate constant of 0.12 min^−1^ (bottom). The quantitative ^13^C NMR spectra resulted in determinations with an accuracy within ±2%, as judged from reproduction and from the signal‐to‐noise ratio in the spectra.

To gain insight into the stability of the carbohydrates and their degradation to organic acids, we continued to follow the time‐resolved reaction progress catalyzed by MgCl_2_‐700/N_2_ (0.38 equiv.) at 120°C. The quantification of the carbohydrates as a function of time showed that the equilibration between glucose, fructose, and mannose proceeded with a kinetics that can be well described as a first order kinetics with a first order rate constant of 0.12 min^−1^ at 120°C (Figure 3). Maximum yields of fructose were reached at 60 min of reaction. The conversion of glucose and other carbohydrates continued, however, due to the accessibility of slower, competing pathways toward the acids shown in Scheme 2. Using MgCl_2_‐700/N_2_ and anaerobic reaction conditions by purging the reactor vial with nitrogen to minimize byproduct formation, the yield for fructose reached just above 40% under these optimized conditions that ensure anaerobic catalyst formation and reaction progress. Hence, the procedure resulted in an equilibration between glucose, fructose, and mannose with a selectivity above 88% and less than 5% byproduct formation during the first hour of the reaction, where 40% yield of fructose was reached according to qNMR determinations.

The effect of different reaction temperatures on glucose isomerization was further probed, using a constant loading of catalyst and temperatures that varied between 100 and 130°C. Unsurprisingly, the formation of degraded and hence entropically favored by‐products within 1 h of reaction time increased with temperature (Figure S4). By contrast, selectivity could be further improved at temperatures below 120°C, resulting in the notable absence of detectable byproducts in the isomerization of glucose to fructose.

### 2.2 Mechanism

**FIGURE 4:**
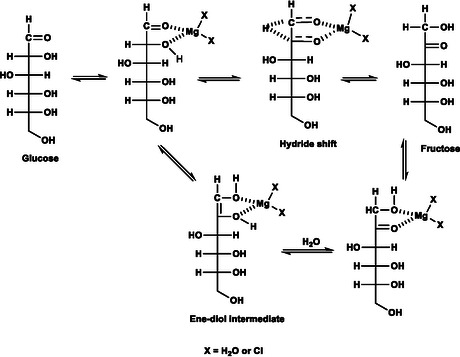
Plausible mechanisms for the routes of glucose‐to‐fructose conversion in water catalyzed by MgCl_2_‐700/N_2_. The reaction can plausibly proceed via 1,2‐hydride shift (top) or via an enediol pathway in the Lobry de Bruyn–Alberda–van Ekenstein transformation.

Upon identifying side reactions catalyzed by basic oxides and upon optimization of selectivity by limiting the presence of basic oxides, we devised a strategy to clarify the reaction mechanism of the glucose‐to‐fructose isomerization by Mg^2+^ salts. This mechanism had remained somewhat controversial [23]. We therefore pursued an isotope tracking study to clarify the relative contribution of different conceivable pathways in the isomerization, either by direct Lewis acid catalyzed 1,2‐hydride shift from the C2 to the C1 position in glucose to yield fructose, or by two steps of tautomerization proceeding via an enediol intermediate in the Lobry de Bruyn–Alberda–van Ekenstein transformation. These pathways are shown in Figure 4. A recent DFT study had suggested that the Mg^2+^ catalyzed glucose‐to‐fructose isomerization predominantly proceeds via the hydride shift mechanism [41]. We employed [2‐^2^H]glucose as the substrate in protonated water (H_2_O) to evaluate the fate of the H‐2 position in glucose and the provenance of the prochiral hydrogens at the C1 position in fructose, either deriving from H‐2 or solvent.

**FIGURE 5:**
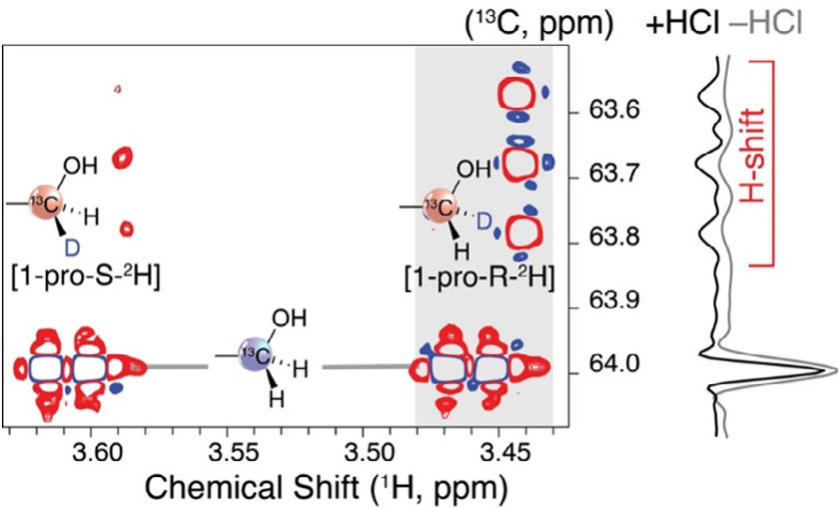
^1^H‐^13^C HSQC showing fructose isotopologues and isotopomers formed from [2‐^2^H]glucose as the substrate in H_2_O. Reaction conditions: 0.02 g (1 equiv.) of [2‐^2^H]glucose, 0.0125 g (0.47 eq) of MgCl_2_‐700/N_2_ (2.5% MgCl_2_‐700/N_2_ as per 0.5 mL of H_2_O), 0.5% HCl (v/v) of a 37% (w/v) stock solution. 120ºC, 0.5 mL H_2_O as a solvent and stirring for 1.30 h in microwave at 120°C. A comparison of the propensity for 1,2‐hydride shift in the absence of added HCl (gray) and in its presence (black, larger fraction of hydride shift) is shown via projections from ^1^H‐^13^C HSQC spectra between 3.48 and 3.43 ppm.

Figure 5 displays the different isotopic composition (isotopologues) and isotopic isomers (isotopomers) resulting from the conversion of [2‐^2^H]glucose in H_2_O, as evidenced using a multiplicity‐edited ^1^H‐^13^C HSQC without employing ^2^H decoupling to distinguish CH and CH_2_ groups at the C1 position of fructose via splitting patterns, the sign of the signal, and isotope effects on the chemical shift. The reaction mixture indicates that most of the fructose is not formed by a direct transfer of the ^2^H from C2 in glucose, as the fructose isotopologue with 2 protons at C1 dominates (signal near 64 ppm, Figure 5, gray projection). While only 26% of the fructose was formed via 1,2‐hydride shift in the absence of added acid, 74% of the fructose incorporated ^1^H from the solvent at the C1 position via an enediol intermediate, even when using MgCl2‐700/N_2_ to limit the presence of basic oxides. Among the 26% of fructose formed via 1,2‐hydride shift, a preference for transfer of the ^2^H into the *pro*‐R position was observed. This preference for the *pro*‐R position relative to the *pro*‐S position was ≈70% to 30%. Preference for 1,2‐hydride shift to the *pro*‐R position reflects the enzymatic process, albeit the preference is less pronounced in the current reaction system than for the 1,2‐hydride shift using Cr(III) or Al(III) catalysis [41].

Considering the large contribution of enediol‐based Lobry de Bruyn–Alberda–van Ekenstein transformation, we hypothesized that general base catalysis may contribute in addition to the Lewis acidity of Mg^2+^, as witnessed by some isomerization via 1,2‐hydride shift. We therefore chose to include 0.5% conc. HCl to the reaction and found that HCl affected speciation of the catalytically active species and poisoned the isomerization to some extent [42, 43]. In the presence of 0.5% conc. HCl, the pH of the reaction mixture prior and after reaction was near pH 3 and isomerization was slowed, consistent with a role of Brønsted basicity in the glucose isomerization. However, isomerization still proceeded, further reducing the propensity to form byproducts (Figure S5). The importance of the 1,2‐hydride shift mechanism under these conditions was again probed using [2‐^2^H]glucose as the substrate in H_2_O (Figure 5). The fraction of fructose formed via 1,2‐hydride shift increased from 26% in the absence of added HCl to 59% in the presence of 0.5% conc. HCl, as witnessed by the population of fructose with deuteriation at the C1 position after using [2‐^2^H]glucose substrate. Thus, the presence of small amounts of HCl was found to both affect the mechanism and the propensity to form byproducts from glucose under MgCl_2_ catalysis. When conducted with protonated glucose in ^2^H_2_O in order to pursue an independent assay for determining the contribution of 1,2‐hydride shift and enediol‐pathways, the majority (≈75%) of the isomerization proceeded via enolization and incorporation of H at the C1 position of fructose in the absence of added HCl (Figure S6), thus corroborating that the Lobry de Bruyn–Alberda–van Ekenstein transformation predominates for glucose‐to‐fructose isomerization in the absence of added Brønsted acid, even if calcination of MgCl_2_ and reaction were conducted under anaerobic conditions.

### 2.3 Comparison to Other Lewis Acid Halides

**FIGURE 6:**
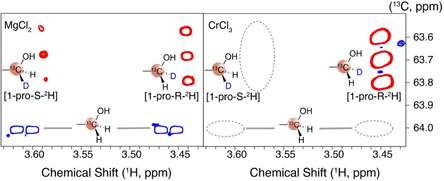
Comparison of ^1^H‐^13^C HSQC showing fructose isotopologues and isotopomers formed from [2‐^2^H]glucose as the substrate in H_2_O using MgCl_2_ (700/N_2_ 0.38 equiv) or CrCl_3_ (0.058 equiv.) as the catalyst. Reaction conditions are those of Figure 5. The assays visualizes that the 1,2‐hydride shift is more pronounced relative to enediol‐based Lobry de Bruyn–Alberda–van Ekenstein transformation and more stereoselective for CrCl_3_ than for MgCl_2_.

Both homogeneous and heterogeneous Lewis acidic sites have been widely used to isomerize glucose to fructose in water, where speciation of the active sites has been experimentally difficult. Similarly, the stereoselectivity of the reaction, as evidenced by ^1^H‐^13^C HSQC assays with reactions using [2‐^2^H]glucose as the substrate in H_2_O, has been rarely reported. We therefore undertook a comparison of stereoselectivity and of the response of active sites for the MgCl_2_ reaction with AlCl_3_ and CrCl_3_‐catalyzed reactions. While glucose to fructose isomerization proceeded both via hydride shift and the Lobry de Bruyn–Alberda–van Ekenstein transformation under MgCl_2_ catalysis, more than 95% of the glucose‐to‐fructose isomerization at comparable conditions proceeded via 1,2‐hydride shift for the CrCl_3_‐catalyzed reaction (Figure 6) and the previously studied AlCl_3_ catalyzed [41] reaction. The stereoselectivity of the latter two reactions was more than 170‐fold in favor of a stereoselective transfer of the hydrogen from C2 of glucose into the *pro‐*R position at the C1 of fructose when using AlCl_3_, while no transfer into the *pro‐*S position was observed in the CrCl_3_‐catalyzed reaction, hinting at a stereoselectivity in the experiment above 200:1. By contrast, this selectivity was only ≈2.5:1 when using MgCl_2_‐700/N_2_ as the catalyst. These observations are consistent with a less rigid complexation of glucose by MgCl_2_ as compared to the transition metal Cr(III) and the post‐transition metal Al(III). The stereoselectivities imply that the transfer into the *pro‐*S position encounters a more than 16 kJ mol^–1^ higher free energy of activation than the corresponding transfer into the *pro‐*R position, both for AlCl_3_·6H_2_O and CrCl_3_·6H_2_O catalyzed glucose to fructose isomerization. By contrast, the transfer into the *pro‐*S position encounters a less than 3 kJ mol^–1^ higher free energy of activation than the corresponding transfer into the *pro‐*R position when using MgCl_2_‐700/N_2_ as the catalyst. Table 1 compares MgCl_2_‐700/N_2_, AlCl_3_·6H_2_O, and CrCl_3_·6H_2_O for their catalytic function in glucose‐to‐fructose isomerization.

**TABLE 1:** Comparison of various chloride for their catalytic function in glucose‐to‐fructose isomerization. Column heads with citations refer to published data, as do table entries with citation, while other data were determined herein.

Catalyst	Activation energy^a^	Mechanism	Stereoselectivity	Inhibition by 1% formic acid	p*K*a^b^
MgCl_2_‐700/N_2_	57–63 kJ/mol	mixed	≈2.5:1	100%	11.2
AlCl_3_·6H_2_O	108–112 kJ/mol	>95% 1,2H shift [41]	≈170.1 [41]	81%	5.0
CrCl_3_·6H_2_O	59–64 kJ/mol	>95% 1,2H shift	>200:1	45%	3.7

a Literature values taken from ref. [22].

b Literature values taken from ref. [44].

### 2.4 Speciation

**FIGURE 7:**
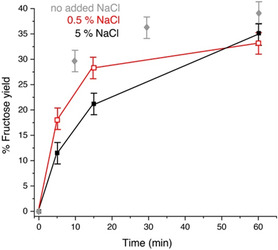
Time course of fructose formation by MgCl_2_‐700/N_2_ as determined by quantitative ^13^C NMR in the presence of 0.5% (red) and 5% (black; both m/v) NaCl. These data argue against an activating role of chloride coordination to the magnesium ion. Reaction conditions: 0.1 g (1 equiv., 0.55 mol/L concentration) of glucose, 0.025 g (0.47 equiv., 0.21 mol/L concentration) of MgCl_2_‐700/N_2_, 1 mL solvent (0.9 mL H_2_O, 0.1 mL D_2_O) and stirring for variable time as indicated in a microwave reactor at 120°C, in the absence and in the presence of 0.5% and 5% (m/v) added NaCl.

**SCHEME 3:**
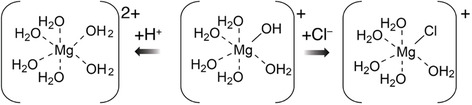
Schematic representation of cations formed upon dissolving MgCl_2_ in aqueous solution. The influence of chloride and proton addition on population shifts according to Le Chatelier's principle is indicated.

Finally, we set out to experimentally evaluate the most plausible candidates for active complexes in the MgCl_2_‐catalyzed glucose‐to‐fructose isomerization in aqueous solution. Again, some controversy exists in the literature on the relative importance of partly hydrolyzed species and on the importance of complexes with chloride [22, 45]. The plausible speciation of chlorides of Lewis acid salts is shown in Scheme 3. Calcination could plausibly support the formation of partly hydrolyzed species (Scheme 3, middle) [39] and rationalize these species as active forms, consistent with classic work on an analogous CrCl_3_ system, while mass spectrometric data had hinted at the presence of chloride‐ligated adducts in solutions of calcinated MgCl_2_, leading to suggestions about their relevance in catalysis. We initially noted that addition of HCl to above 0.5% largely inhibited the activity of MgCl_2_‐700/N_2_. According to Scheme 3, addition of protons and chloride both will depopulate the partly hydrolyzed species shown in the middle of Scheme 3. Hence, the deactivation by HCl supported a role of this species or a similar one. For further support, we systematically attempted to affect the speciation by adding chloride without acid and acid without chloride. When adding up to 5% NaCl to a MgCl_2_‐700/N_2_ catalyzed reaction without other changes, we observed a slowing of the reaction, as evident in the experimental data of Figure 7. Hence, we conclude that chloride coordination to MgCl_2_ is an unlikely candidate for the active species.

**FIGURE 8:**
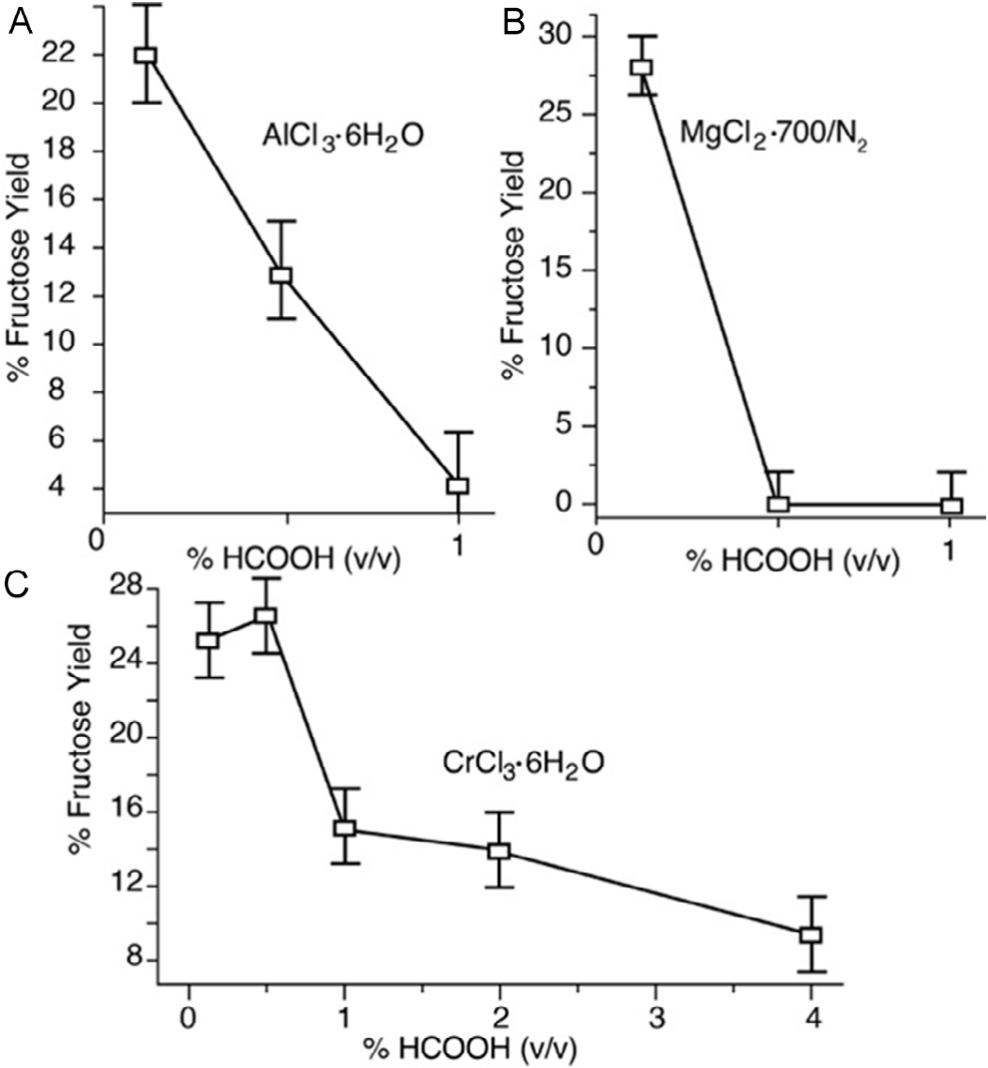
Inhibitory effect of various concentrations of added formic acid on glucose‐to‐fructose isomerization by (A) Al^3+^, (B) Mg^2+^ and (C) Cr^3+^. Reaction conditions: 0.1 g (1 equiv., 0.55 mol/L concentration) of glucose, 1 mL aqueous solvent and stirring for 30 min (to avoid equilibration) in a microwave reactor at 120°C. Reactions catalyzed by Mg^2+^, Al^3+^, and Cr^3+^ were conducted using 0.47, 0.058, and 0.058 equiv., respectively.

Due to the formation of organic acids as byproducts in the MgCl_2_‐700/N_2_ catalyzed glucose‐to‐fructose isomerization and in many biomass converting reactions, and due to the ambition to shift speciation by protonation without adding halides, we added formic acids at various concentrations to the MgCl_2_‐700/N_2_ catalyzed reaction. Formic acid was inhibitory for the glucose‐to‐fructose isomerization, consistent with a central role of partially hydrolyzed species like the one in Scheme 2 (middle) for catalytic activity. The equivalent species had been suggested as the active species in previous studies on CrCl_3_‐catalyzed reactions that ultimately lead to acidic products [45], and we therefore evaluated the inhibitory effect of formic acids also on Cr(III) and Al(III)‐catalyzed glucose‐to‐fructose conversions. Also the Cr(III) and Al(III)‐catalyzed reactions were somewhat obstructed by the addition of formic acid, albeit to a lower degree than the MgCl_2_‐700/N_2_‐catalyzed isomerization. The differential behavior is consistent with decreasing p*K*a values for Mg^2+^, Al^3+^, and Cr^3+^, that imply the need of lower pH values and higher acid content to depopulate the partly hydrolyzed form in the order Mg^2+^, Al^3+^, and Cr^3+^. Accordingly, the addition of 1% formic acid abolished detectable activity for MgCl_2_‐700/N_2_ catalyzed isomerization, while 19% and 55% of the activity were retained for the Al^3+^ and Cr^3+^‐catalyzed reactions, respectively (Figure 8).

## 3 Conclusion

In conclusion, we show that MgCl_2_ is a viable catalyst for converting glucose to fructose in water with excellent selectivity if competing pathways leading to C—C bond breakage are considered. Improved selectivity can be achieved when using MgCl_2_ that was calcined and used in inert atmosphere to limit the presence of basic oxides. Opposite to theoretical predictions, both 1,2‐hydride shift and isomerization via an enediol intermediate are experimentally found to contribute to the isomerization by such material. Mildly acidic conditions could tune the relative importance of 1,2‐hydride shift and isomerization via an enediol intermediate, while affording selective reactions predominantly hinging on the Lewis acidity of Mg^2+^. Careful experimentation to study effects of mineral and organic acids, as well as chloride ions, pointed to partially hydrolyzed hydrated forms as the active species in Mg^2+^ catalysis, and similar trends were found for Al^3+^ and Cr^3+^ species. Thus, qNMR measurements of activity in conjunction with titrations by additives provided valuable insight on the active catalyst species, while multiplicity‐edited HSQC spectra clarified the stereoselectivity of 1,2‐hydride shifts in the substrate. Here, considerable differences were found for the preference of hydride shift into the *pro‐*R position, while transfer to the *pro‐*S position encounters more than 16 kJ mol^–1^ higher barriers in Al^3+^ and Cr^3+^ catalysis, but less than 3 kJ mol^–1^ higher for Mg^2+^ catalysis. A plausible explanation for this behavior could be differences in the rigidity of complexes between glucose and the metal ion, leading to strongly different selectivities. Overall, we find that diligent catalyst preparation can lead to highly selective conversions using naturally abundant salts, while diligent experimentation can clarify similarities and differences in the mechanism and active species of Mg^2+^, Al^3+^, and Cr^3+^ catalyzed glucose‐to‐fructose isomerization. Anion effects warrant further investigation, for instance considering that ligands are known to critically tune activities in glucose‐to‐fructose isomerization [46], considering that MgCl_2_ has previously been considered to function as a chloride anion source in the conversion of glucose via fructose to HMF [47] and considering a recent interest in MgBr_2_ rather than MgCl_2_ as the catalyst in glucose‐to‐fructose isomerization [23]. As we find that precise control over speciation is key for highly selective biomass conversion, design of selective glucose‐to‐fructose isomerization catalysts inspired by the methods and findings provided herein could focus on rationally tuning the Mg^2+^ environment to pursue enzyme mimesis, while recognizing that excess basicity and leaching may promote side reactions.

## 4 Experimental Section

### 4.1 Chemicals

D‐Glucose (96%) and D‐galactose was purchased from Sigma Aldrich (St. Louis, MA, USA) and D‐mannose (99%) and D‐xylose (99%) were purchased from Merck (Darmstadt, Germany). D‐[2‐^2^H]glucose was purchased from Omicron biochemicals (South Bend, IN, USA). Lewis acidic salts MgCl_2_ (99%), MgBr_2_ (99%), and MgI_2_ (99%) were likewise purchased from Alfa Aesar and used without further purification. CrCl_3_·6H_2_O and AlCl_3_·6H_2_O were purchased from Merck (Darmstadt, Germany). HCl was used as a 37% (w/v) solution (Fisher, Waltham, MA, USA). Deuterated water (D_2_O) was purchased from Deutero GmbH (Kastellaun, Germany). Deionized (DI) water was obtained from a Millipore water purifier system (Merck Millipore, Burlington, MA, USA) and used for sample preparations and dilutions.

### 4.2 Calcination

All magnesium halides were calcined at 700°C in a muffle furnace for 4 h. MgCl_2_ was additionally calcined at 700°C in a muffle furnace under N_2_ atmosphere for 4 h.

### 4.3 General Reaction Procedure

The typical reaction procedure consisted of weighing glucose (100 mg, 1 equiv.) and MgCl_2_‐700/N_2_ catalyst (20 mg, 0.38 equiv.) into a microwave vial and adding 0.9 mL deionized (DI) water as well as 0.1 mL D_2_O prior to closing the microwave cap and stirring at room temperature for 1 min to dissolve the glucose. Subsequently, the reaction mixture was heated to 120°C or other desired temperatures in a Biotage (Uppsala, Schweden) initiator plus Microwave reactor for 1 h. PH values were measured prior to and after the completion of the reaction using a Mettler Toledo pH meter. The procedure was repeated in the presence of added NaCl to evaluate the effect of halide species, specifically MgCl^+^ forms [22], on the reaction speed. In the presence of added NaCl (up to 5% m/v), a reduction in the initial rate of glucose‐to‐fructose conversion was observed.

To compare the mechanism and activity of various Lewis acids, the procedure was repeated using MgCl_2_, CrCl_3_·6H_2_O (5.8 mol%) or AlCl_3_·6H_2_O (5.8 mol%) to convert 100 mg glucose in 1 mL water at 120°C for 30 min. Various concentrations of formic acid were added as shown in Figure 8, prior to quantification of isomerization.

The time‐dependent conversions, yields, and selectivities in the reactions were calculated as

$$\%\;\mathrm{conversion}=\frac{\left(C_{\text{reactant},t=0}-C_{\text{reactant}}\right)}{C_{\text{reactant}},t=0}\times 100$$

$$\%\;\mathrm{yield}\;\mathrm{of}\;\mathrm{product}\;k=\frac{C_{k}}{\left(C_{\text{reactant},t=0}\right)}\times 100$$

$$\%\;\mathrm{selectivity}\;\mathrm{of}\;\mathrm{product}\;k=\frac{C_{k}}{\left(C_{\text{reactant},t=0}-C_{\text{reactant}}\right)}\times 100,\text{respectively.}$$

### 4.4 X‐ray Diffraction (XRD)

Powder XRD experiments were conducted on a Malvern Panalytical Empyrean diffractometer, equipped with a 1Der X‐ray detector, using Cu K‐*α* (1.5406 Å) radiation in reflection mode. Samples were measured between 3.5 and 90° 2*θ* with a step size of 0.008 2*θ* at a scan speed of 10.8 2*θ*/s. XRD profiles prior and post calcination are compared in Figure S2, indicating a stable framework, but minor structural changes during calcination, consistent with previous observations for MgCl_2_ describing sequential dehydration and hydrolysis steps along rather complex pathways [39].

### 4.5 NMR Samples and Spectroscopy

For ex situ ^13^C NMR experiments, the reaction mixture was typically transferred to a 5 mm NMR sample tube. Reactions employing isotope‐labeled D‐[2‐^2^H]glucose were performed on a scale of 40 mg of glucose (1 equiv.) and 8 mg of MgCl_2_‐700/N_2_ (0.38 equiv.) using 3 mm NMR sample tubes. NMR spectra were acquired on an 800 MHz Bruker (Fällanden, Switzerland) Avance III instrument equipped with an 18.7 T magnet and a 5 mm TCI cryoprobe or on a 400 MHz Bruker Avance III HD instrument equipped with a 9.4 T Ascend magnet and a 5 mm Prodigy BBO 400 S1 probe. Quantitative ^13^C NMR spectra were used for quantifications and were acquired using the zgig30 pulse sequence and using an inter‐scan relaxation delay of 60 s on the 18.7 T magnet, as were ^1^H‐^13^C HSQC and HMBC assignment spectra. The quantitative ^13^C NMR spectra resulted in determinations of conversion, yield, and selectivity with an accuracy within ±2%, as judged from reproduction and from the signal‐to‐noise ratio in the spectra. Potentially different responses of isotopologue species with respect to their signal recovery in ^1^H‐^13^C HSQC spectra were calibrated by comparison to quantitative ^1^H‐^13^C HSQC for conversions of [1‐^13^C]glucose in D_2_O. Multiplicity‐edited ^1^H‐^13^C HSQC spectra were acquired with a 20 ppm spectral width in the ^13^C dimension centered around a carrier offset of 69 ppm upon isomerizing D‐[2‐^2^H]glucose. Multiplicity‐edited ^1^H‐^13^C HSQC spectra were acquired as data matrices of 1024 times 256 complex data points in the direct and indirect dimensions, respectively, sampling the FID for 160 ms and 64 ms in the ^1^H and ^13^C dimensions, respectively.

### 4.6 Data Analysis

All NMR data were acquired and processed with zero filling to twice the number of acquired data points, and subsequently analyzed using Bruker Topspin 3.5 pl6. Integrals were plotted in pro Fit 7 (QuantumSoft, Zurich, Switzerland).

## Supporting Information

NMR spectra showing the effect of magnesium halide; detailed XRD data; identification of dihydroxyacetone and other byproducts formed using MgCl_2_; NMR spectra showing the effect of temperature on byproduct formation; effect of low concentrations of HCl to neutralize basicity; isotopologues formed when converting protonated glucose in deuterated water; spectra showing the inhibitory effect of formic acid on isomerization by Mg^2+^ and Cr^3+^. Additional supporting information can be found online in the Supporting Information Section. **Supporting Fig. S1:** Comparison of the product mixture obtained when using MgCl_2_ (top) or MgI_2_ (bottom) calcined at 700°C as the catalyst. Reaction conditions: 0.1 g (1 equiv., 0.55 mol/L concentration) of glucose, 0.38 equiv. of MgCl_2_‐700 or MgI_2_‐700, 1 mL solvent (0.9 mL H_2_O, 0.1 mL D_2_O), 1 h at 120^o^C (Microwave reactor with stirring). **Supporting Fig. S2:** XRD patterns for MgCl_2_ and MgCl_2_‐700/N_2_, showing a stable framework. However, differences in intensity and fine structure at 2θ = 15.21º and 49.24º, respectively, were observed, indicating minor alterations to the material after calcination at 700^o^C under N_2_ atmosphere. **Supporting Fig. S3:** (A) Glucose conversion for 10 min at 120°C using MgCl_2_‐700/N_2_ catalyst shows the formation of dihydroxyacetone as an intermediate. A ^1^H‐^13^C HSQC spectrum of the reaction mixture is shown in red, and a spectrum for authentic dihydroxyacetone is overlayed in gray. ^1^H‐^13^C HMBC on the reaction mixture validates the assignment through a ^2^J_CH_ correlation between the alcohol proton and the ketone group. Reaction conditions: 0.1 g (1 equiv., 0.55 mol/L concentration) of glucose, 0.38 equiv. of MgCl_2_‐700/N_2_, 1 mL solvent (0.9 mL H_2_O, 0.1 mL D_2_O), 10 min at 120°C (Microwave reactor with stirring). (B) 1D ^13^C spectrum acquired using [1‐^13^C]glucose as the substrate and showing that the C1 position was predominantly converted to formic acid, or it was converted to the C1 position of metasaccharinic acids, the C2 position of glycolic acid and acetic acid, as well as the C1 and C3 position of lactic acid. **Supporting Fig. S4:** Glucose isomerization at different reaction temperatures, showing higher tendency for byproduct formation (15% carbon balance) at higher temperatures, as expected. Reaction conditions: 0.1 g (1 equiv., 0.55 mol/L concentration) of glucose, 0.38 equiv. of MgCl_2_‐700/N_2_, 1 mL solvent (0.9 mL H_2_O, 0.1 mL D_2_O), 1 h at varying temperatures (Microwave reactor with stirring). **Supporting Fig. S5:** (A) Glucose isomerization after at 120°C by MgCl_2_‐700/N_2_ catalyst in the absence (blue) and in the presence (red) of 0.5% concentrated HCl. Reaction conditions: 0.1 g (1 equiv., 0.55 mol/L concentration) of glucose, MgCl_2_‐700/N_2_, 1 mL aqueous solvent, 120°C (Microwave reactor with stirring). The reaction was conducted for 1 h in the absence of added HCl, and for 1.5 h in the presence of added HCl. Byproducts in the absence of added HCl are highlighted by asterisks. (B) Inhibition of the reaction at addition of more than 0.5% concentrated HCl. **Supporting Fig. S6:** (A) ^1^H‐^13^C HSQC showing the formation of C1‐deuterated fructose in the conversion of protonated [1‐^13^C]glucose in ^2^H_2_O. (B) Quantitative ^13^C NMR spectrum indicating that the majority (≈75%) of the isomerization incorporated 2H at the C1 position of fructose. **Supporting Fig. S7:** Glucose isomerization by MgCl_2_ (47.2 mol%) or CrCl_3_·6H_2_O (5.8 mol%) in the presence of various formic acid content (0.12 ‐ 4% v/v). Reaction conditions: 0.1 g (1 equiv., 0.55 mol/L concentration) of glucose, MgCl_2_‐700/N_2_ or CrCl_3_·6H_2_O, 1 mL solvent (0.9 mL H_2_O, 0.1 mL D_2_O) containing various formic acid content as indicated, 30 min. Addition of formic acid elicits slower isomerization in both instances.

## Funding

This study was supported by Villum Fonden (Grant 57925).

## Conflicts of Interest

The authors declare no conflicts of interest.

## Supporting information

Supplementary Material

## Data Availability

The data that support the findings of this study are available from the corresponding author upon reasonable request.
