# Supplementary material for: Selective and Tunable Routes for Glucose to Fructose Conversion Using MgCl2 Catalysis and Comparison to Other Metal Ions
Source: ChemistryOpen. 2026 Feb 1;15(2):e202500495. doi: 10.1002/open.202500495 (PMC12862016; doi:10.1002/open.202500495)
Supplement: Supplementary file 1 — Supplementary Material [file OPEN-15-e202500495-s001.pdf]

Selective and Tunable Routes for Glucose to Fructose Conversion Using $MgCl_2$ Catalysis and Comparison to Other Metal Ions

Ramesh Maragani and Sebastian Meier*

Department of Chemistry, Technical University of Denmark, Kemitorvet, Building 206, 2800 Kgs Lyngby, Denmark

Number of pages: 8

Number of Figures: 7

Contents:

Figure S1. ^{13}C NMR spectra of isomerization with different magnesium halides S1

Figure S2. XRD patterns for $MgCl_2$ and $MgCl_2$-700/N_2 S2

Figure S3. Spectra showing products from competing reactions S3

Figure S4. Glucose isomerization at different reaction temperatures S4

Figure S5. Effect of the presence of 0.5% HCl and higher concentrations S5

Figure S6. Formation of C1-deuterated fructose in ^2H_2O S6

Figure S7.Inhibitory effect of formic on various catalysts S7

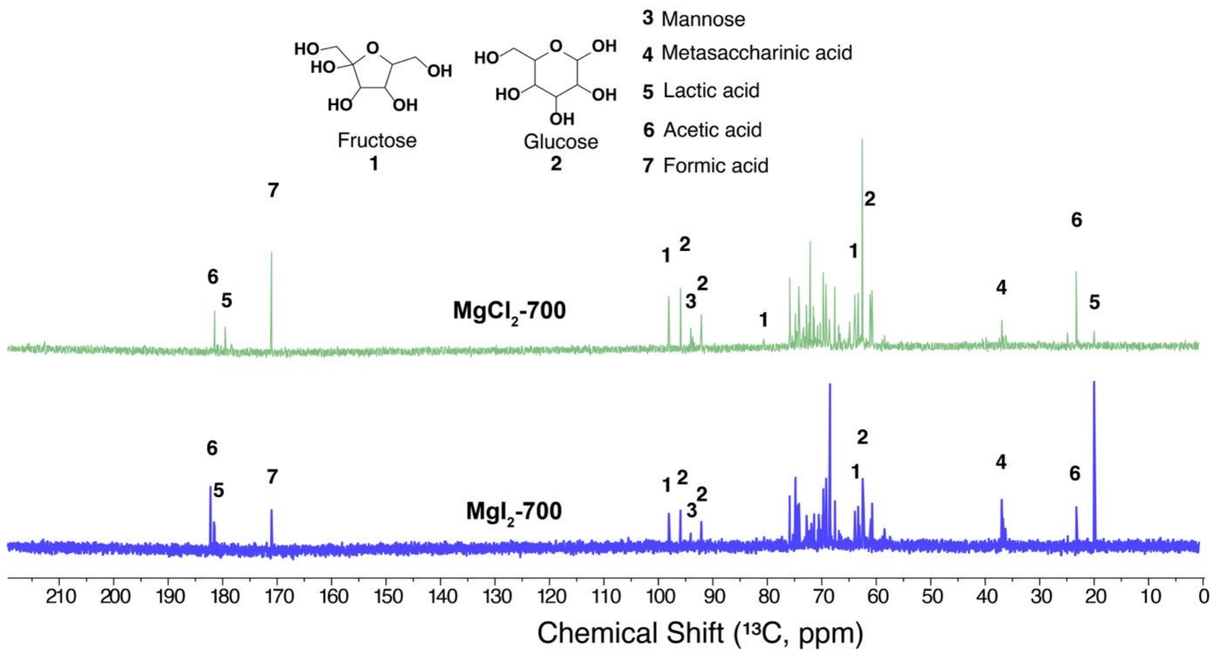

Figure S1. Comparison of the product mixture obtained when using $MgCl_2$ (top) or MgI_2 (bottom) calcined at 700 ºC as the catalyst. Reaction conditions: 0.1 g (1 equiv., 0.55 mol/L concentration) of glucose, 0.38 equiv. of $MgCl_2$-700 or MgI_2-700, 1 mL solvent (0.9 mL H_2O, 0.1 mL D_2O), 1 h at 120 ºC (Microwave reactor with stirring).

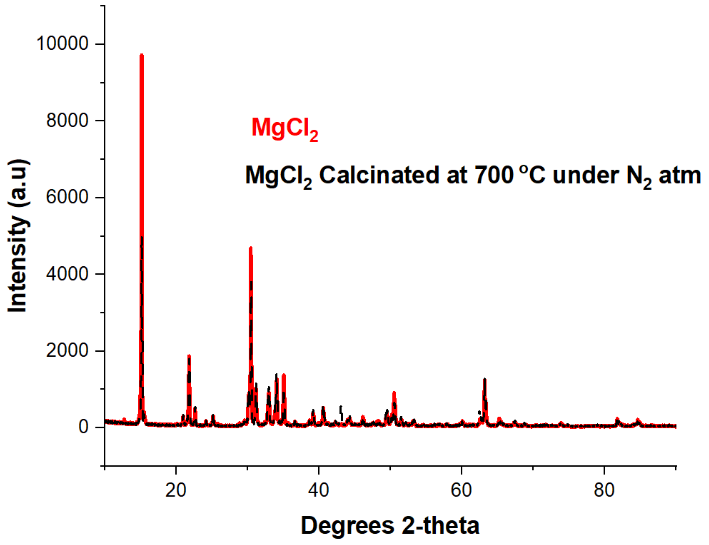

Figure S2. XRD patterns for $MgCl_2$ and $MgCl_2$-700/N_2, showing a stable framework. However, differences in intensity and fine structure at 2θ = 15.21° and 49.24°, respectively, were observed, indicating minor alterations to the material after calcination at 700 °C under N_2 atmosphere.

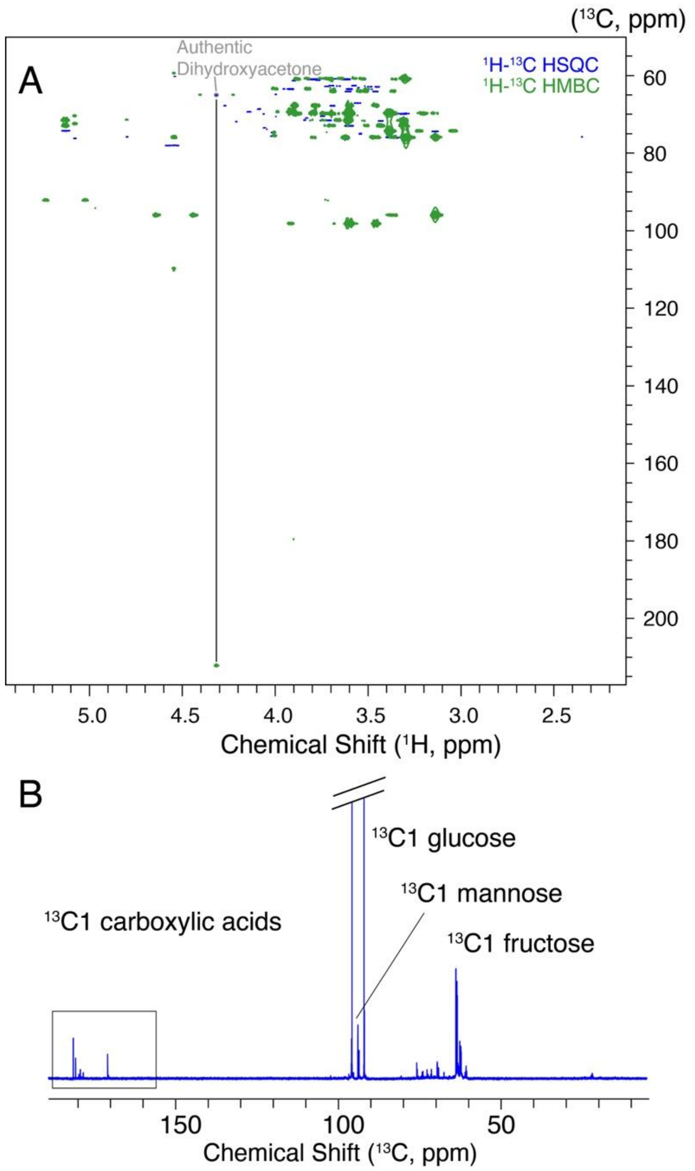

Figure S3. (A) Glucose conversion for 10 min at 120 ºC using $MgCl_2$-700/N_2 catalyst shows the formation of dihydroxyacetone as an intermediate. A ^1H-^{13}C HSQC spectrum of the reaction mixture is shown in red, and a spectrum for authentic dihydroxyacetone is overlayed in grey. ^1H-^{13}C HMBC on the reaction mixture validates the assignment through a $^2J_{CH}$ correlation between the alcohol proton and the ketone group. Reaction conditions: 0.1 g (1 equiv., 0.55 mol/L concentration) of glucose, 0.38 equiv. of $MgCl_2$-700/N_2, 1 mL solvent (0.9 mL H_2O, 0.1 mL D_2O), 10 min at 120 ^oC (Microwave reactor with stirring). (B) 1D ^{13}C spectrum acquired using [1-^{13}C]glucose as the substrate and showing that the C1 position was predominantly converted to formic acid, or it was converted to the C1 position of metasaccharinic acids, the C2 position of glycolic acid and acetic acid, as well as the C1 and C3 position of lactic acid.

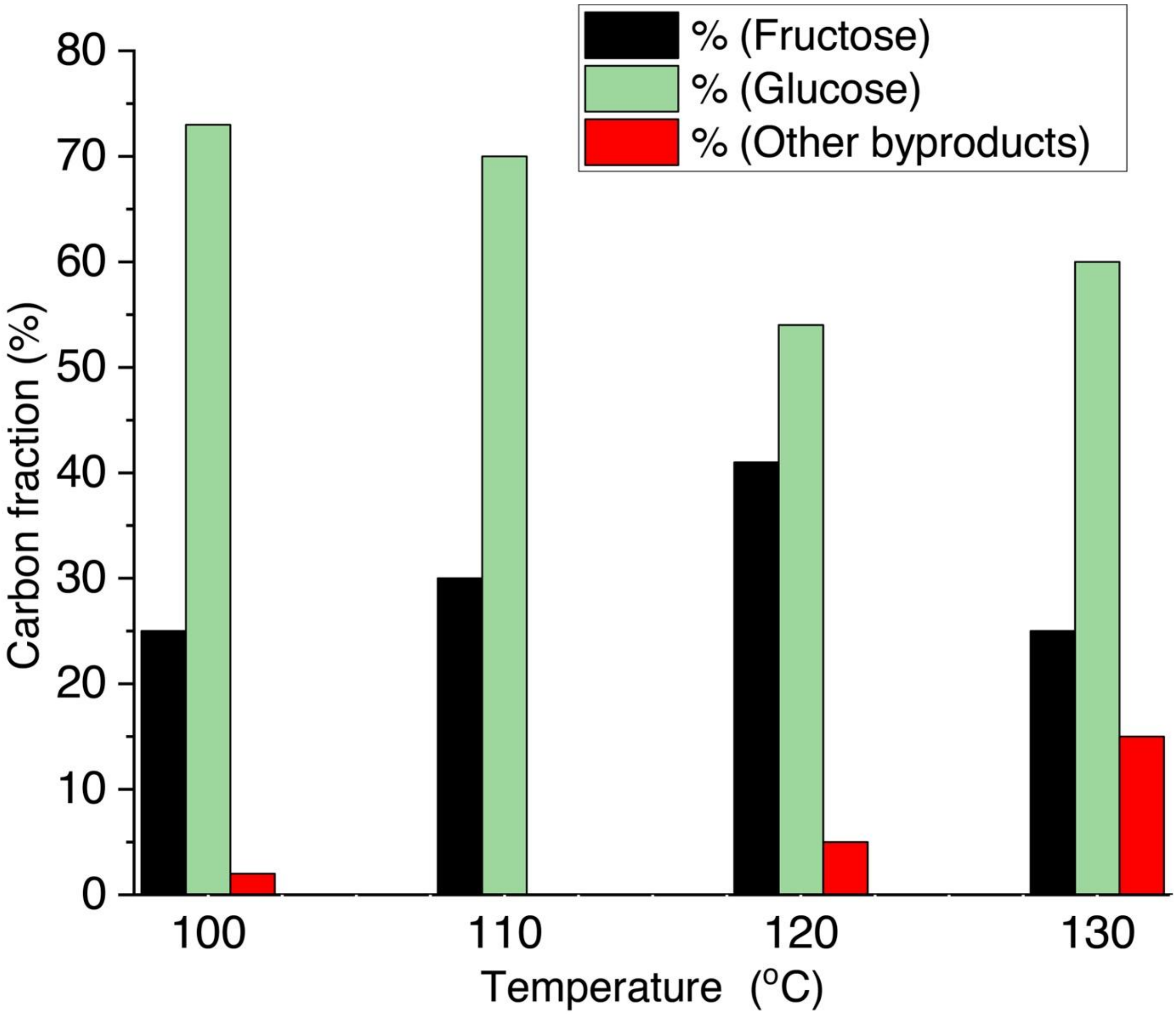

Figure S4. Glucose isomerization at different reaction temperatures, showing higher tendency for byproduct formation (15% carbon balance) at higher temperatures, as expected. Reaction conditions: 0.1 g (1 equiv., 0.55 mol/L concentration) of glucose, 0.38 equiv. of $MgCl_2$-700/N_2, 1 mL solvent (0.9 mL H_2O, 0.1 mL D_2O), 1 h at varying temperatures (Microwave reactor with stirring).

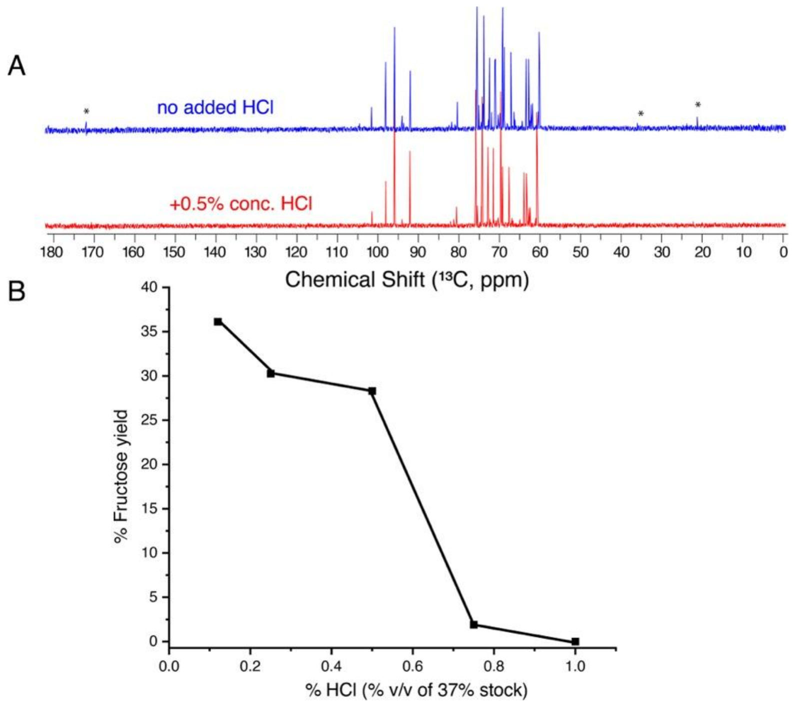

Figure S5. (A) Glucose isomerization after at 120 ºC by $MgCl_2$-700/N_2 catalyst in the absence (blue) and in the presence (red) of 0.5% concentrated HCl. Reaction conditions: 0.1 g (1 equiv., 0.55 mol/L concentration) of glucose, $MgCl_2$-700/N_2, 1 mL aqueous solvent, 120 ^{o}C (Microwave reactor with stirring). The reaction was conducted for 1 h in the absence of added HCl, and for 1.5 h in the presence of added HCl. Byproducts in the absence of added HCl are highlighted by asterisks. (B) Inhibition of the reaction at addition of more than 0.5% concentrated HCl.

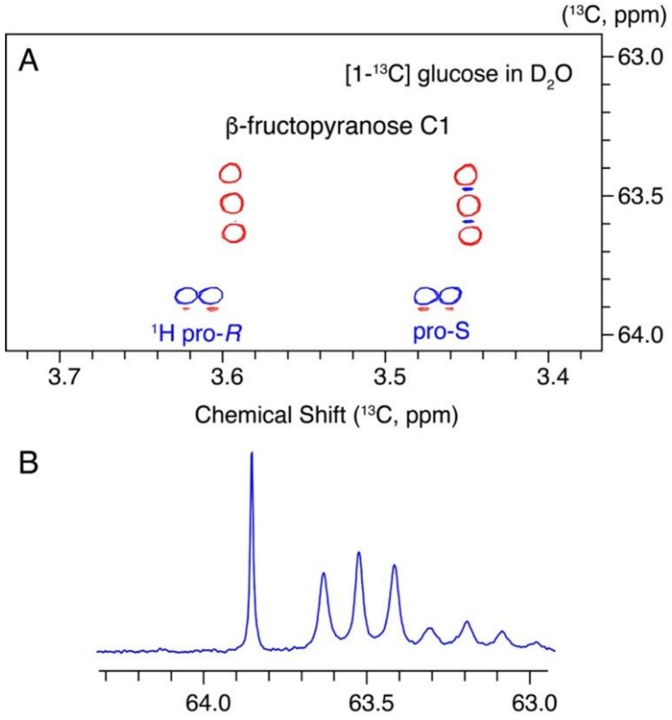

Figure S6. (A) ^{1}H-^{13}C HSQC showing the formation of C1-deuterated fructose in the conversion of protonated [1-^{13}C]glucose in $^{2}H_2O$. (B) Quantitative ^{13}C NMR spectrum indicating that the majority (~75%) of the isomerization incorporated ^{2}H at the C1 position of fructose.

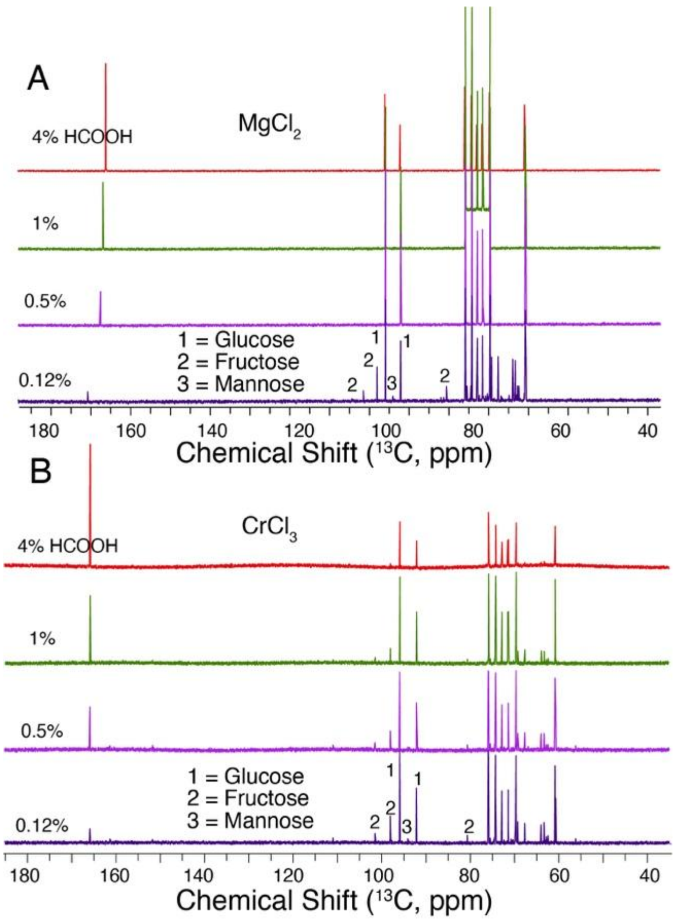

Figure S7. Glucose isomerization by $MgCl_2$ (47.2 mol%) or $CrCl_3 \cdot 6H_2O$ (5.8 mol%) in the presence of various formic acid content (0.12 - 4% v/v). Reaction conditions: 0.1 g (1 equiv., 0.55 mol/L concentration) of glucose, $MgCl_2$-700/N_2 or $CrCl_3 \cdot 6H_2O$, 1 mL solvent (0.9 mL H_2O, 0.1 mL D_2O) containing various formic acid content as indicated, 30 min. Addition of formic acid elicits slower isomerization in both instances.
